# Sensitivity analysis for enhancing crude oil recovery with continuous flow gas lift: A study in reference to the porous media of the upper Assam basin, India

**DOI:** 10.1016/j.heliyon.2023.e17466

**Published:** 2023-06-27

**Authors:** Dhrubajyoti Neog

**Affiliations:** Department of Petroleum Technology, Dibrugarh University, Assam, 786004, India

**Keywords:** Well performance, Vertical lift performance, Gas injection optimization, Sensitivity analysis, Continuous flow gas lift, Reservoir pressure

## Abstract

Despite minimal formation damage or water-cut, the majority of oil wells in brown oil fields cease to flow naturally. The current study looks into and analyses what caused a self-flowing well in the upper Assam basin to become non-flowing. The non-flow condition of the well was investigated in the current work as a function of water cut, reservoir pressure, reservoir rock permeability, and GOR. The effect of WHP and WHT on these functions was investigated. This work incorporates innovative methodology that uses the PROSPER simulation model for assessing the possibility of establishing flowability in a dead well based on inflow (IPR) and vertical lift performance (VLP). Subsequent analysis was carried out to examine the scope of producing this dead well under continuous flow gas lift. For this, the current work first examined the tubing diameter and reservoir temperature as standalone parameters to find out if they have any role to play in the flowability of the dead well. Following this, sensitivity analysis was done taking four parameters into account, i.e., reservoir pressure, reservoir rock permeability, water cut, and total GOR. In the current work, surface equipment correlation was established using Beggs and Brill correlation, while vertical lift performance was established using correlation from Petroleum Expert. The results of the current work highlight that a well's production rate under continuous flow gas lift can be enhanced by employing an optimised gas injection rate. The findings of this work conclude that higher reservoir pressure enables an oil well to produce with a high water cut under a continuous flow gas lift system, provided there are no formation damage issues on the well.

## 1 Introduction

Low productivity in ageing oil fields is an unavoidable matter of concern for the oil industry. The gas lift offers a wide range of solutions for increasing production rates from naturally depleted oil wells. Gas lift systems, when properly installed and optimised, improve the well production performance of any naturally flowing oil well. It is vital to analyse the techno-economic feasibility of installing an artificial lift in order to ensure that production can be sustained at a profit. Reservoir pressure drop and excessive water cuts are two of the most common oil well issues, both of which usually result in a decrease in output. Wells that produce crude oil based on the natural energy in their reservoirs can benefit from artificial lift systems. The most prevalent artificial lift methods used in the oil industry to maintain crude oil production in naturally flowing wells are Gas Lift (GL), and Pumping (Electrical Submersible Pump (ESP), Sucker Rod Pump (SRP), Hydraulic Pump (HP), and Progressive Cavity Pump (PCP)). The most common artificial lift systems are ESP and GL, both of which may greatly increase oil production. When applied to wells producing at high GLR, however, ESP has limitations, but GL is suited for such conditions because it can reuse the gas dissolved in oil to improve production rate.

Gas lift is a well-known and well-proven method of oil extraction. The gas lift lowers the bottom hole pressure, allowing more fluid to flow into the well, and hence more pressure is drawn down between the reservoir and the bottomhole. Gas lift design comprises establishing depths as well as the location of valve installations to achieve gas injection based on the flow regime existing inside the tubing. The two types of gas injection are tubing flow and annular flow. The basic method of gas lifting by continuous flow is to continually inject gas into the well, which lightens the fluid column and displaces the oil, with the injected gas at the valve setting depth. The intermittent lifting method of gas lifting is used in wells with a low productivity index [1]. Continuous gas injection is used in the first approach. This process is simple to set up but requires more gas to be injected into the well, whereas the latter just requires intermittent and small volumes of gas [2]. Gas is injected at a certain position in the tubing of a production well. By maintaining a low bottomhole flowing pressure and a high-pressure difference between the reservoir and the bottomhole flowing pressure, lift gas is able to transfer fluid to the top of the wellhead. When gas reaches the inside of the tubing, it lightens the fluid column, allowing the fluid to flow freely along the tubing. Injecting too much gas, on the other hand, may raise the bottomhole pressure in the reservoir, lowering the rate of oil production. Because the gas phase moves faster than the liquid phase, too much gas injection causes gas slippage, which causes less liquid to flow into the tubing [3]. Injection optimization is a solution to this issue that not only increases oil production but also aids in the distribution of the required optimal amount of gas injection to various wells. Moreover, the amount of gas needed for different water-cut wells differs. Following the optimization of the injected gas rate and volume, wells with varying water cuts can be helped in improving oil recovery by enabling cost-effective distribution of the injection gas to the reservoirs [4]. In addition, a non-flowing or underperforming producing well with a low production rate can be revived or improved by injecting lift gas into it [5]. However, before putting a well on gas lift, a production optimization analysis should be carried out to estimate the maximum cumulative oil output that the well is capable of producing using the gas lifting production mechanism [6]. As cumulative oil production increases, the reservoir pressure decreases and, as a result, the plateau production rate decreases. Pumping and gas lifting are examples of artificial lift procedures that aid in the recovery of brown oil fields. A multitude of factors influence the artificial lift selection criteria for oil wells, including reservoir and fluid properties, water cut, well and reservoir pressure. The inflow performance relationship (IPR) and tubing performance relationship (TPR) determine the well's ability to flow naturally or with artificial lift.

The productivity of a previously shut down oil well with continuous flow gas lift is enhanced when the issue of non-flowing is the low reservoir pressure; otherwise, the flow rate may not be higher than the well's natural flow rate. As a result, the findings contradict the widely held belief that gas lifting aids in increasing crude oil production in naturally flowing wells [7]. It has been observed in some case studies that increasing gas injection does not result in increased oil production. Wells that can produce under current conditions, have good productivities, no substantial water cuts, and low reservoir pressure can flow without the need for increased gas injection rates [8]. When it comes to dealing with pressure loss problems or taking on the challenges of producing heavy oil from oil wells, all three artificial lift technologies (continuous flow gas lift, intermittent flow gas lift, and electrical submersible pump) are competitive. The simulation tool PROSPER (Production and System Performance Analysis System) is effective in optimising gas lift operating parameters and predicting comparative well performance for each of the artificial lifts [9]. This tool is found to be extensively in use for studies related to gas lift design and to find out under what conditions the self-flowing state of a naturally flowing well can be sustained and which method of artificial lift would be feasible to produce in the future. For this, the fluid flow study in the reservoir simulator has to be based on realistic and accurate grid types, which may vary for different reservoir types. E.g., for fracture models, elastic gridding is preferred over conventional [10]. To study well performance analysis with a simulator for a heterogeneous formation, it is primarily necessary to define well geometry for each productive zone. A fine zonation is considered a strategy for integrated well performance analysis that takes into account addressing the heterogeneity issue of the reservoir formation based on a simulation study [11].

There hasn't been much research into determining the efficiency of gas lift systems. The attached compressor unit, the piping system utilised in the transportation of gas from the gas lift plant to the receiver well, the well's performance, and the efficiency of pipes connecting the gas lift well to the metering station are all factors in the gas lift system's performance [12]. One of the common issues, i.e., paraffin and wax deposition, may arise when the lift gas reacts with the well fluid in the tubing. During gas lifting, it creates bubbles that lighten the oil column, changing the pressure, temperature, and fluid composition in the process. Depending on the wax appearance temperature of the oil in the tubing, a drop in crude oil temperature causes the production of wax deposits. This difficulty encountered during the gas lift process, however, can be addressed by allocating an optimum gas injection rate. In addition, the well's output is determined by the rate of gas injection, which can be adjusted [13]. In addition to single-string completion, dual-string completion entails injecting gas into two independent strings and optimising the split factor for injected gas dispersion at a ratio between the two strings of tubing. However, there is a dearth of research on dual-string gas lift production at present. It's difficult to design gas lifts in twin completion wells, and it can lead to gas robbing. A phenomenon known as "gas robbing” occurs when one string receives a lot of gas while the other is low. As a result, the production rates of both tubing strings are frequently reduced [14]. Installing gas lift valves with tubing macaroni, in addition to the usual gas lifting procedure, is a cost-effective way to produce from a well that does not flow naturally. This solution saves the operator money because, otherwise, a workover to install a new tubing mandrel with gas lift valves would normally be required [15]. Recently, research was conducted to develop a hybrid artificial lift model that combines electrical submersible pumping and gas lift. This method has the potential to enhance output rates while reducing the amount of energy consumed by the ESP system [16].

To increase crude oil production, gas lift optimization is essential, and the depth and rate of gas injection are two of the most significant variables in the optimization assessment. In the case of continuous flow gas lift, economic optimization of lift gas and its cycle efficiency may increase profit by saving injection gas and improving the efficiency of oil recovery from mature oil fields [17]. Wellhead pressure (WHP), tubing dimensions, producing gas oil ratio (GOR), water cut, and gas injection rate are all production parameters that can be optimised to enhance oil production [18].

**Fig. 1:**
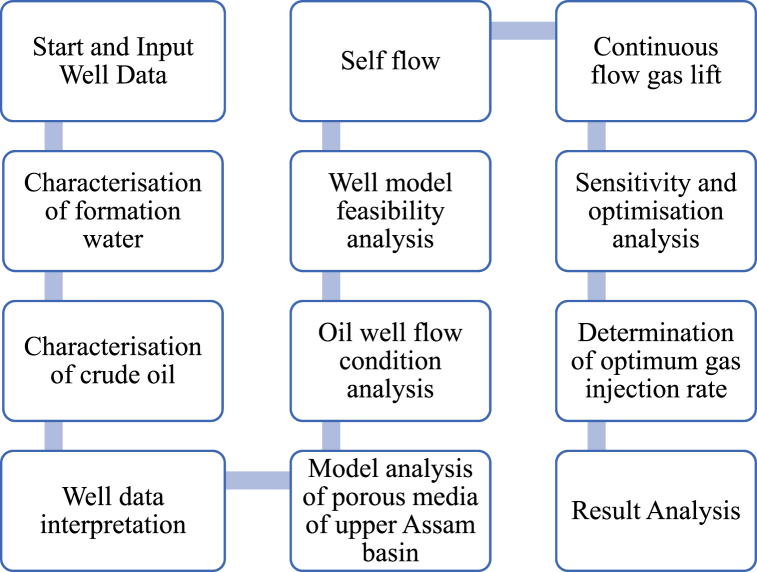
Work design for modal analysis.

The current study presents a novel approach to investigate the feasibility of enhancing oil recovery with the installation of a continuous flow gas lift system with consideration of a combination of factors that include reservoir fluid, rock, and well parameters in the non-flowing porous media of the upper Assam basin. This study first discusses the methodology and materials that were used to characterise the reservoir fluid and the porous media in the upper Assam basin. This followed the performance analysis of the non-flowing natural porous media using production modelling with the PROSPER simulator. Following this, the current study proposes a model for the porous media in the upper Assam basin to find out the scope of starting up the production in the oil well with the installation of a continuous flow gas lift system. The subsequent sections of the present work provide a detailed study, taking into account the oil well parameters that need to be optimised to sustain flowability in the natural flow. Finally, the current work presents a discussion and draws conclusions based on the results of sensitivity and optimization analysis that highlight the success of continuous flow gas lift installation in enhancing oil recovery in the porous media of the upper Assam basin. The study design on the assessment of the natural flow porous media to sustain and enhance flow with the addition of the gas lift installation using model analysis with PROSPER is shown in Fig. 1.

## 2 Materials and methodology

### 2.1 Materials

**Fig. 2:**
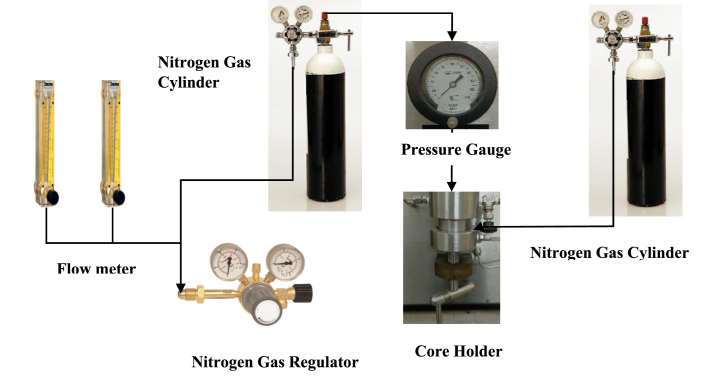
Air permeameter set up for measurement of permeability.

This study used the porous medium of a non-flowing primary energy-driven oil well in the upper Assam basin to quantify porosity and permeability. An Ofite Model 360 Air Permeameter from Houston, Texas was used to assess the absolute permeability of the sandstone reservoir rock. The TPI-219 helium porosimeter from Coretest Systems, INC. was used to assess the porosity of non-flowing porous media in the upper Assam basin. In the current analysis, the crude oil used was a wellhead sample from the upper Assam basin oil well. The obtained well fluid was first processed in the separating funnel and then centrifuged for separation of the water component in order to determine the distinct physical properties of the obtained crude oil sample. The density, °API, pour point, and specific gravity of the crude oil sample were then determined and are presented in Table 1. The physical and petrophysical properties of the sandstone porous medium were measured and are shown in Table 2 [19,20]. **As indicated in** Table 1**, the formation water separated from the crude oil samples was then characterised.** Fig. 2 illustrates the air permeameter setup used to assess the permeability of sandstone porous media in the upper Assam basin. The current work undertook model analysis using the PROSPER simulator to develop first a model of a naturally flowing reservoir, which was then analyzed using PVT data, reservoir and fluid data, and well data. The associated information for naturally flowing porous media was then employed to create a gas lift installation model to examine the efficacy of the gas lift model in a similar situation to enable fluid flow in the porous media. Finally, the efficacy of the well in flowing under continuous flow gas lift installation was evaluated based on the inflow and vertical lift performance observed for the well under study. Based on the laboratory data related to solution GOR, oil gravity, gas gravity, density, and formation water salinity, the PVT match was done in the PROSPER software. For measurement of PVT properties, this work used the black oil model. Beggs-Brill and Petroleum Expert 2 were found to have the best correlation to determine the vertical lift performance (VLP). The findings with VLP account for pressure regime change with changing reservoir pressure, bottom hole flowing pressure, and tubing pressure losses. Due to the change in flow regime while flowing through the vertical tubing, multiphase flow is obtained; however, a detailed study on this aspect is not within the scope of the model analysis in the current work.

**Table 1:** Reservoir fluid properties.

Parameters	Measured Values
Crude oil	
Density	848.9 kg m^−3^
°API	35
Pour Point, °C	32
Specific gravity	0.8498
Formation water	
Salinity	3100 ppm
pH	6.4
Water cut (%)	20

**Table 2:** Petrophysical properties of sandstone rock.

Core ID	Diameter, D in.	Permeability, k md	Average permeability, k md	Porosity, φ (%)	Rock Density, ρ g cm^−3^	Rock Type RT
C1	1	299		0.1975	2.64	Sandstone
C2	1	298		0.1860	2.63	Sandstone
C3	1	304	300	0.2120	2.60	Sandstone
C4	1	295		0.1756	2.59	Sandstone
C5	1	303		0.2037	2.58	Sandstone

### 2.2 Methodology

#### 2.2.1 Determination of permeability

The sandstone porous media from the upper Assam basin was first plugged into a into a total of five number of 1 inch diameter core plugs, which were then water washed and cleaned at the Soxhlet apparatus with a 50:50 solvent mixture of toluene and methanol. The core plugs were immersed in the solvent solution, and the solvent combination was gently heated in the Dean-Stark apparatus at 40 °C. The core samples were cleaned for 72 h, following which the colour of the reflux solution was detected to be a clear liquid. After cleaning the samples, they were transferred to an ultrasonic cleaner, where the cleaned core plugs were agitated at room temperature for around 1 h. The ultrasonic cleaning procedure was performed to remove any surface impurities that had not been removed by the Dean-Stark device. The sandstone plug was then dried for 72 h in a hot-air oven to ensure total dryness in the porous media, thereby ensuring that the rock medium was devoid of liquid contaminants. After that, the clean, dry core plugs were placed in the core holder of the Ofite Model 360 Air Permeameter, made in Houston, Texas, in order to determine their absolute permeability. The Ofite Air Perm comes with two flow meters: one for low permeability samples and the other for high permeability sandstone rock measurements. In the experimental work, nitrogen gas was passed through the core plugs during the analysis, and the flowmeter data was recorded versus applied nitrogen gas pressure. Each of the flowmeters has a total of 150 measurements, starting at 0 and increasing by 10 scale points. The injection gas pressure was steadily increased in the experimental study, with readings taken at 10-point intervals on an upward scale until the maximum reading of 150 was reached. The nitrogen source pressure in the Ofite Air Perm model was set at 200 psi, and the injection gas pressure was gradually increased in the experimental analysis to take readings at all 10 points. The pressure gauge linked to the Air Perm displays the applied nitrogen gas pressure values. In the experimental examination, the basic Darcy's equation was employed to calculate the permeability of each sample, which assumed the flow through core plugs to be a laminar flow. For each injection gas pressure, the observed permeability was then shown to be a reciprocal of the mean gas pressure (1/Pm) at ambient conditions. The observed permeability of the porous media at various gas injection pressures was then used to compute the permeability of the porous media in the upper Assam basin. Table 2 shows the results of the estimated absolute or liquid permeability values.

#### 2.2.2 Determination of water cut

In this experimental analysis, the obtained wellfluid from the upper Assam basin's porous medium was centrifuged following separation in the separating funnel for about 56 h at room temperature. This process enhances gravity segregation based on the density differential between the crude oil sample and the associated water. The volume of the separated water in the measuring cylinder was then measured and the corresponding data for the water cut recorded. The Soxhlet apparatus was then used to put the remaining well fluid sample that had not been separated into oil and water. The Dean Stark method was used to separate the samples in this study. In the round-bottom flask, an identical amount of toluene was introduced as a solvent to the unseparated well fluid sample. The crude oil and toluene mixture were then gently heated in the round-bottom flask. The Soxhlet apparatus was fitted with a graded receiver into which the reflux fluid from the round bottom flask falls. The water content was calculated by subtracting the amount of the accumulated volume of toluene that filled the receiver from the total volume of the collected liquid. The percentage of water cut in well fluid samples determined using the Dean Stark method and gravity separation is shown in Table 1.

#### 2.2.3 Performance analysis

**Fig. 3:**
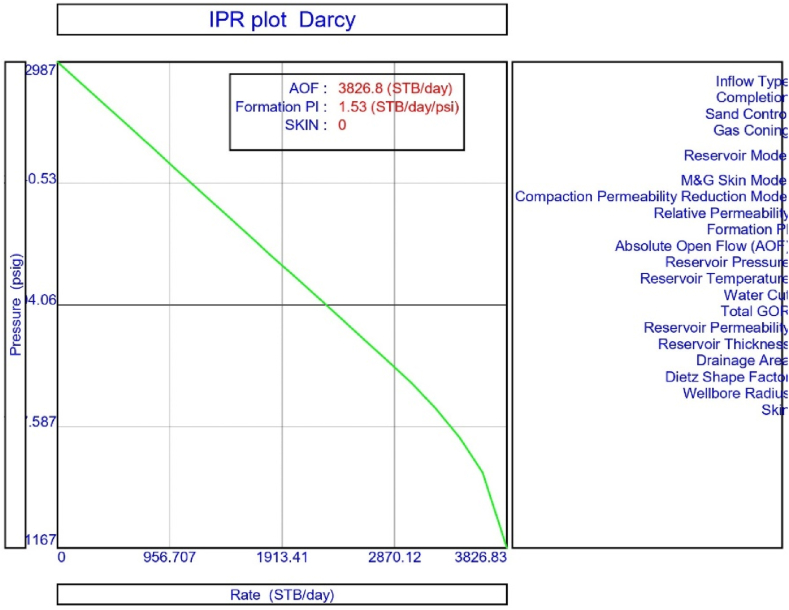
IPR plot for natural flow porous media.

In this study, model analysis was conducted in the PROSPER (PROduction and Systems PERformance) simulator for a non-flowing porous medium using well data that included PVT properties, with a reservoir pressure and temperature of 2987 psi and 172 °F, respectively, as shown in Table 3. The PVT characteristics of oil-producing porous media in the upper Assam basin were assessed using the Black Oil model, which integrates oil and water as the flowing fluid. In the current work, the bubble point pressure (P_b_) was established as 1128.68 psi after the PVT matches were done. The best measured data for the well in the natural flow was then determined using non-linear regression analysis. In the PVT regression study, the Glaso correlation was found to be the best match suitable correlation for the determination of PVT parameters, bubble point pressure (P_b_), gas oil ratio in solutions (Rs), and formation volume factor (β_o_). In this investigation, the Bean et al. correlation was recommended for evaluating oil viscosity. The model analysis work included information on downhole equipment as well as production figures based on both measured data and the true vertical depth of the oil sand. The well model also incorporated the downhole setup data that took into account the tubing and casing internal diameters, as well as the recorded depth data. The input data for analysing and matching the geothermal gradient was then applied to the wellhead and formation depths of 0 and 8500 feet, respectively. In the current analysis, the formation, or perforation depth temperature, was set at 172 °F, while the temperature at the wellhead was set at 90 °F. Following that, the overall heat transfer coefficient was set at 8 BTU/hour/ft^2^ for oil, gas, and water, with average capacities of 0.53, 0.51, and 1 for each. Darcy's reservoir model was used to compute the IPR for a non-flowing natural flow well with a reservoir pressure of 2987 psi, a reservoir temperature of 172 °F, and a water cut of 20% (Table 1). The porous media used in the study had no signs of formation damage, and experimental studies determined that the formation permeability was 300 md (Table 2). In the model analysis, the reservoir thickness was limited to 10 feet, and other details are presented in Table 4. Darcy's reservoir model was used to construct the IPR of the oil sands in the upper Assam basin, which indicates the PI of the natural (self)-flowing well to be 1.53 bbl/day/psi for the absolute open flow rate of 3826.8 STB/day (Fig. 3).

**Table 3:** Data used for matching PVT properties.

Parameter	Value
Solution GOR	200 scf/STB
Reservoir Pressure	2987 psi
Reservoir Temperature	172 °F
Bubble point pressure, P_b_	1128.68 psi
Correlation used	
P_b_, R_s_, β_o_	Glaso
Oil viscosity	Bean et al.
PVT Matched	Yes
PVT Method	Black Oil

**Table 4:** Well data.

Parameters	Values
Downhole data	
Measured Depth, MD, ft	True Vertical Depth, TVD, ft
0	0
Bottom Measured Depth, ft	Bottom True Vertical Depth, ft
8500	8500
Downhole Equipment	
Tubing ID, in.	2.44
Tubing Depth, ft	8490
Casing ID, in.	4.89
Casing Depth, ft	8500

The present study considers the Darcy model to reflect the natural flow of porous media with inadequate aquifer support. The low reservoir pressure in the well is not able to help crude oil production. The assumptions that were used in the current work include the supposition of steady-state flow conditions for the model analysis, which infers a constant pressure boundary condition. Darcy steady state model was applied in the current work to establish the IPR for the well under study. The fluid flow is considered single-phase, with reservoir pressure above bubble point pressure and below it multiphase flow effects being considered. In this study, the reservoir formation is considered to be homogeneous, and it is a single well study that was producing naturally with depletion energy and weak aquifer support. The outer boundary of the drainage area is constant and sealed. Effective permeabilities K_x_, K_y_, and K_z_ are uniform. The thickness H, porosity Ø and permeability are also considered to be consistent. Water influx is on to the well; hence, during cumulative production, the initial reservoir pressure is different from the pressure at the boundary. Above bubble point pressure, the fluid flow is single-phase, while below it, multiphase flow develops owing to depletion drive effects as well as the low water cut in the well under study.

Wellhead oil samples as well as the sub-surface porous media (Table 2) were collected in the current analysis. Reservoir fluid properties for the well under study were evaluated; refer to Table 1. The issue of heavy particles like asphaltene was not reported for the well. However, changing pressure regimes and GOR were considered in the work. The model for the oil well included well data, PVT properties, reservoir fluid properties, as well as the properties of the sub-surface sandstone porous media. In addition, the water cut percentage was taken into account for the analysis with the PORSPER simulation model. Production well data were the basis for the model analysis. Sensitivity analyses were performed for both the non-flowing oil well and the well on gas lift. This work takes into account the pressure regime changes as well as the GOR, in addition to the issues of water cut and tubing diameter. As the primary issue with the well was low reservoir pressure, the simplified Darcy model was used for the inflow performance analysis.

The current study by the PROSPER simulator used empirical correlations developed by petroleum experts that consider flow regime changes when the wellfluid flows from the intake of tubing to the top of the tubing. IPR was generated for the non-flowing well as well as the artificially flowing well (continuous flow gas lift) using Darcy steady state correlation. Different flow regimes for vertical lift performance were evaluated using Wallis and Griffith correlation for bubble flow, Hagedorn and Brown for slug flow, Duns and Ros for transient flow, as well as annular mist flow. Although the wellfluid influx to the wellbore is single-phase, due to pressure depletion, the wellfluid changes flow regime from the intake of tubing to the top of tubing. The different calculations done for pressure drop and the correlations used for examining fluid flow have all been used the way they are supposed to be used in PROSPER. VLP correlations were established in the current work with Petroleum Experts 2. This study uses the original Peng-Robinson equation of state (EOS) to predict PVT properties. The analysis of the well's performance is largely based on production data, and the anisotropy of the reservoir is not computationally studied in this work due to a lack of data regarding this. The well under study is producing from a single zone, and the reservoir inflow model studied was Darcy's. Further included in the model were no skin damage, pressure, temperature, producing GOR, and water cut data. The assessment of the reservoir's shape is not covered in the current work, although completion details have been included.

This work used Darcy steady state model for IPR prediction. The equation of states used for inflow model (IPR) is for a straight line provided the reservoir pressure is above bubble point pressure (Eq. (1)).

[1] $$Q=J\left(P_{s}-P_{b}\right)$$

However, Vogel empirical relationship was used in PROSPER for reservoir pressure below bubble point pressure (Eq. (2)).

[2] $$\frac{Q}{Q_{\max}}=1-0.2\;\left(\frac{P_{wf}}{P_{r}}\right)-0.8\;{\left(\frac{P_{wf}}{P_{r}}\right)}^{2}$$

Composite Vogel inflow solution was used for account of water cut and for the effect of gas formation below bubble point pressure (Eq. (3)).

[3] $$J=\frac{Q}{F_{o}\left\{P_{r}-P_{b}+\frac{P_{b}}{1.8}(1-0.2\frac{P_{wf}}{P_{r}}-0.8{\left(\frac{P_{wf}}{P_{r}}\right)}^{2}\}\right\}+F_{w}\left(P_{r}-P_{wf}\right)}$$

where: Q = flow rate, P_s_ & P_b_ = bubble point pressure, Q_max_ = maximum flow rate, P_wf_ = bubble point pressure, P_r_ = reservoir pressure, F_o_ = oil cut, and J = productivity index.

The PROSPER simulator provides multiple correlation options that are linked with different equations of states. In this work, fluid properties, petrophysical properties, as well as reservoir characteristics were used, which were either measured in the lab or known in the context of sandstone porous media in the upper Assam basin. PVT analysis was done on PROSPER based on input data belonging to upper Assam basin oil well. VLP curves were generated in this study using correlations by Petroleum Experts, which are considered to be accurate for the field condition under study. The inflow performance prediction was done using Darcy steady state method and using Vogel correlation for below bubble point pressure for two-phase as well as composite well conditions. The issue in the studied well is of low reservoir pressure and it is in initial stage of production. Hence, the above-mentioned equations of state are used as implemented in PROSPER, considering them to be feasible for the current well condition. Also PROSPER analysis are governed largely by the production well data which were primarily in use in the current work.

The assumptions that were used in the current work include the supposition of steady-state flow conditions for the model analysis, which infers a constant pressure boundary condition. Darcy steady state model was applied in the current work to establish the IPR for the well under study. The fluid flow is considered single-phase, with reservoir pressure above bubble point pressure and below it multiphase flow effects being considered.

The work used PROSPER which is a simulator used primarily to study the production and system performance analysis work. The porosity and permeability were evaluated in the instruments called Helium Porosimeter and Ofite Model 360 Air Permeameter, respectively. The petrophysical properties in the work were considered to be constant throughout the study.

**Fig. 4(a):**
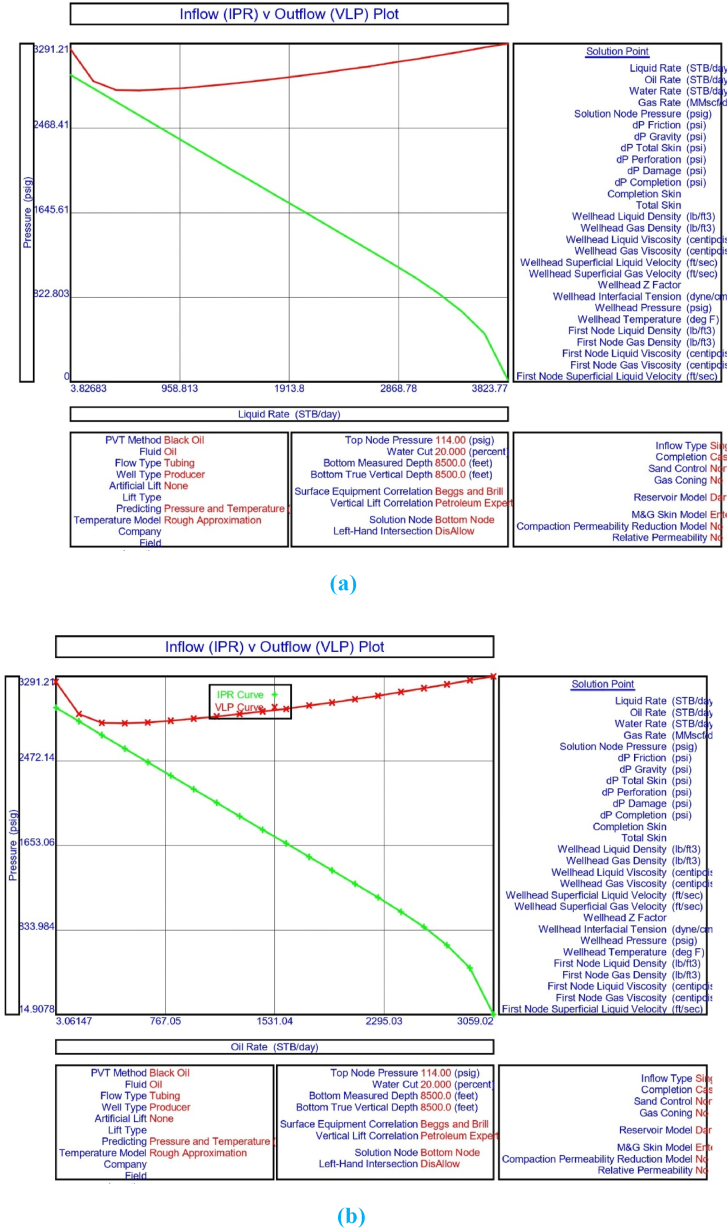
IPR + VLP curve for natural flow well (liquid rate). Fig. 4(b). IPR + VLP curve for natural flow well (oil rate).

The current study examines the viability of producing a dead well under continuous flow gas lift. For this, at first, the dead well was investigated by generating the IPR as well as the VLP of the well. As the IPR versus VLP curve (Fig. 4a and b) showed no intersection point, the dead well condition was established. Following this, the well performance, taking into account the IPR vs. VLP plot, was further studied under a set of conditions, i.e., at various water cuts (Fig. 6a and b), reservoir pressures (Fig. 7a and b), changing reservoir permeability (Fig. 8a and b), and GOR (Fig. 9a and b). In addition, the effect of WHP and WHT on oil flow rate was also examined for different water cuts (Fig. 10), reservoir pressure (Fig. 11), rock permeability (Fig. 12), and GOR (Fig. 13). Table 5, Table 6, Table 7, Table 8 also contain references to the findings. The findings of the current work infer that continuous flow gas lift resulted in flowability of the dead well, as evidenced in Fig. 14 (a) and (b). This work included an optimization study of the injection gas rate for the gas lift operation, as shown in Fig. 16, comparing oil production rate (STB/day) versus gas injected rate (MMScf/day). The valve setting depth for gas lift was figured out. The results are available in Fig. 17, Fig. 18. Like in the natural flow, sensitivity analysis was done for continuous flow gas lift as well. This includes extensive analysis based on the IPR versus VLP plot for CFGL for different well conditions. These conditions are initially considered standalone parameters with respect to tubing diameter (Fig. 19a and b) and reservoir temperature (Fig. 20a and b). Following this, the conditions are combined on a single well, i.e., reservoir pressure, sandstone rock permeability, water cut, and total GOR (Fig. 21a and b). Likewise, the effect of WHP and WHT on flow rate under CFGL was also studied first as an individual parameter for tubing diameter (Fig. 22) and reservoir temperature (Fig. 23). The data on the optimization of the gas injection rate can be studied in Table 9. Sensitivity findings are also available in Table 10, Table 11, Table 12.

**Fig. 5:**
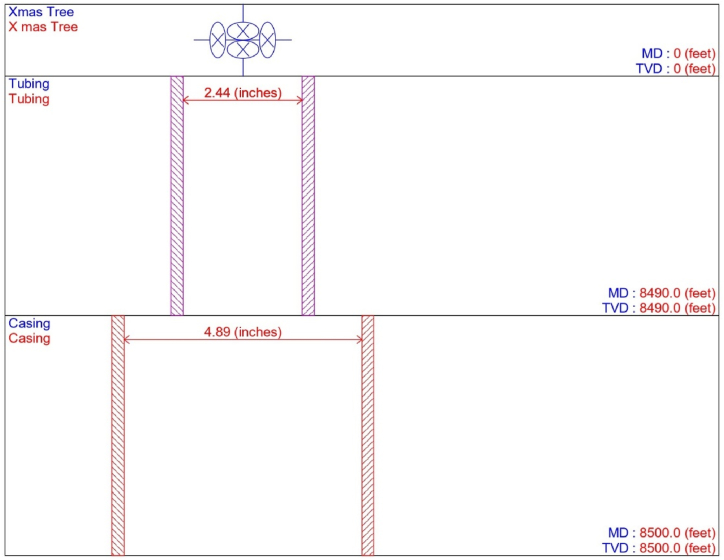
Well completion set up.

**Fig. 6 (a):**
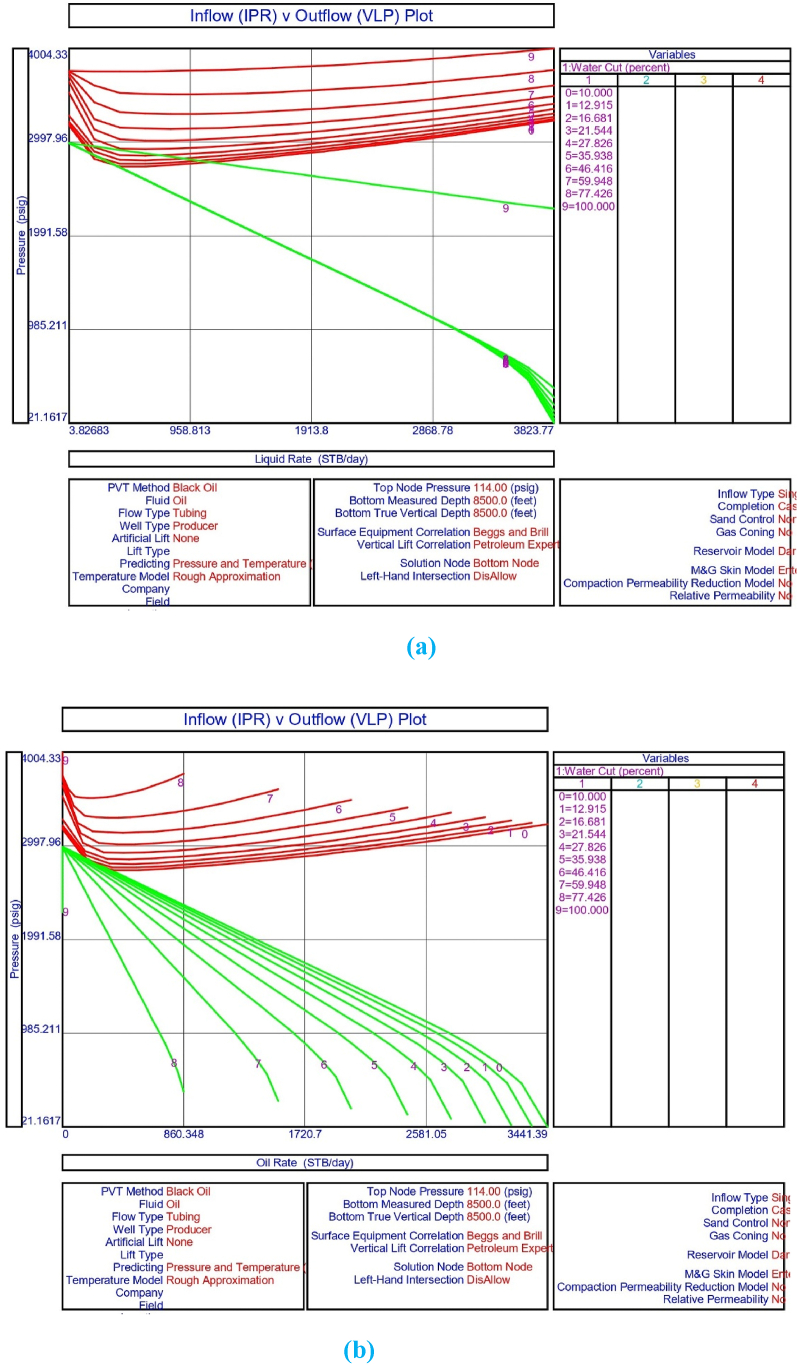
Sensitivity analysis for natural or self-flow well at different water cuts. Fig. 6(b). Sensitivity analysis on production flow rate of natural or self-flow well at different water cuts.

**Fig. 7(a):**
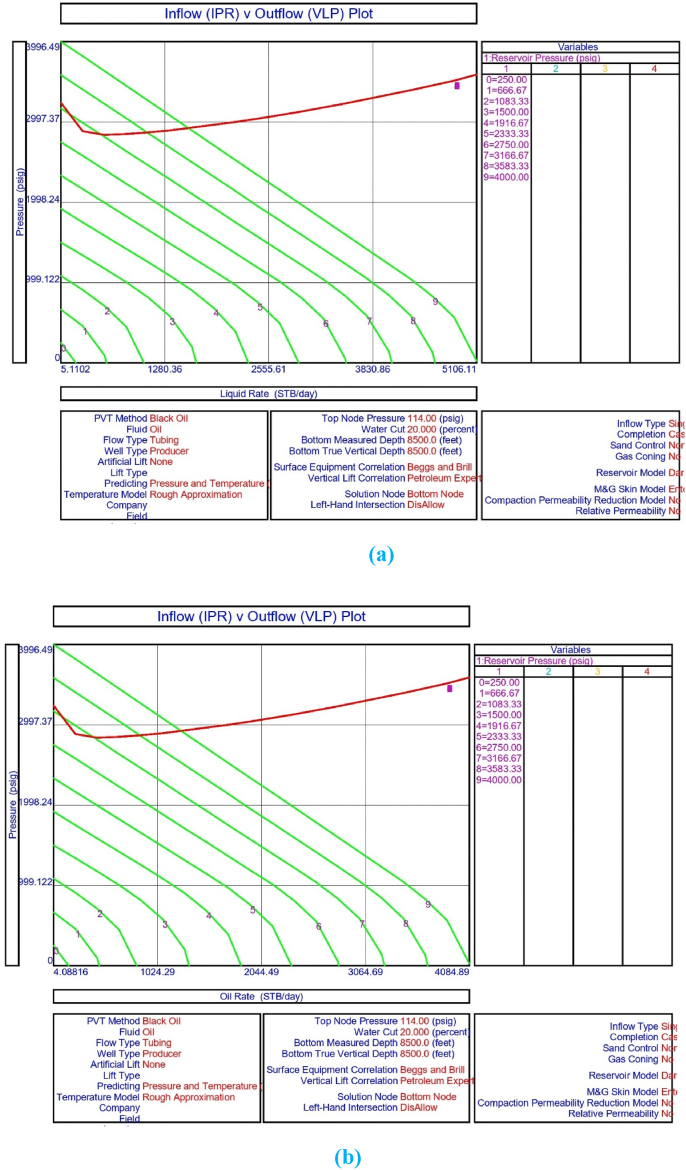
Sensitivity analysis on production flow rate of natural or self-flow well at different reservoir pressures (liquid rate). Fig. 7 (b). Sensitivity analysis on production flow rate for natural or self-flow well at different reservoir pressures (oil rate).

**Fig. 8 (a):**
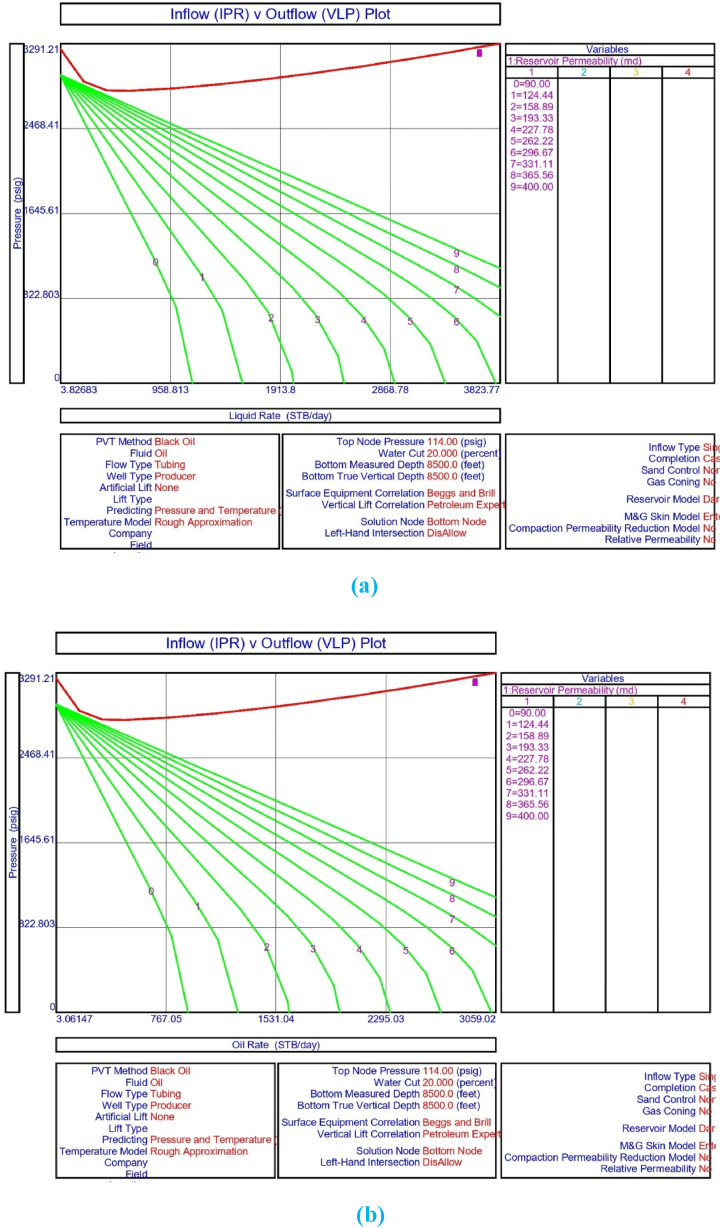
Sensitivity analysis on production flow rate for natural or self-flow well at different reservoir permeabilities (liquid rate). Fig. 8 (b). Sensitivity analysis on production flow rate for natural or self-flow well at different reservoir permeabilities (oil rate).

**Fig. 9 (a):**
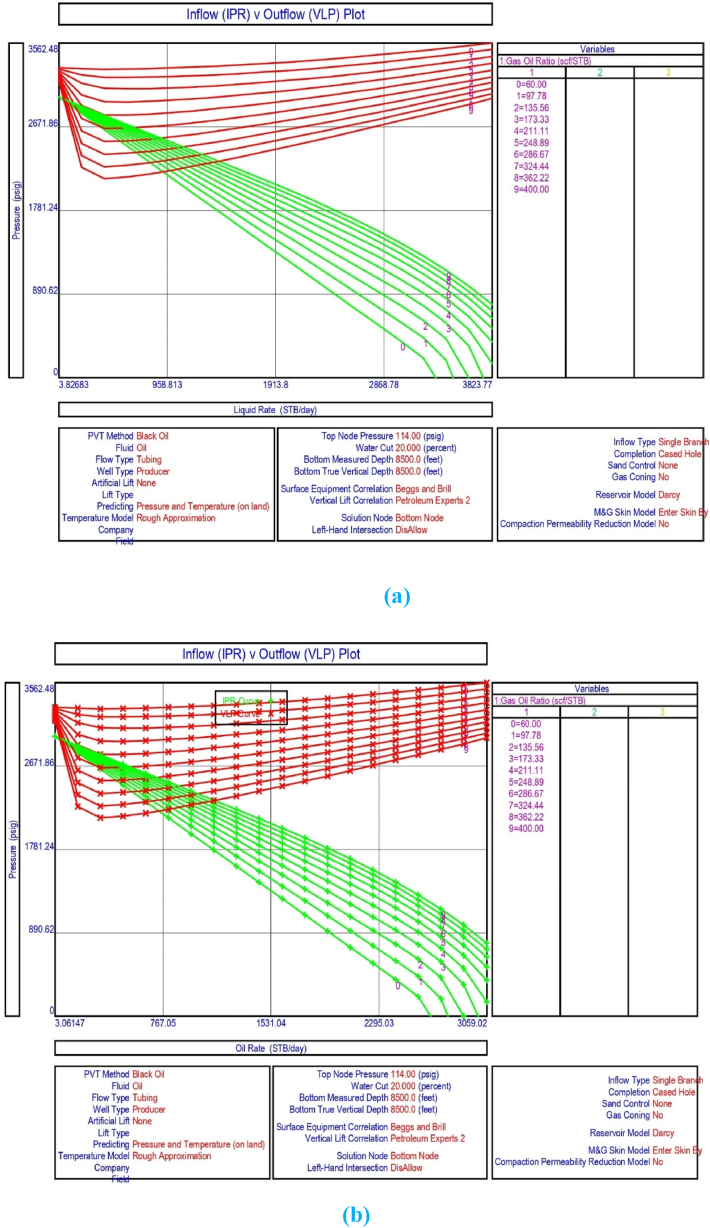
Sensitivity analysis on production flow rate for natural or self-flow well at different GOR (liquid rate). Fig. 9 (b). Sensitivity analysis on production flow rate for natural or self-flow well at different GOR (oil rate).

**Fig. 10:**
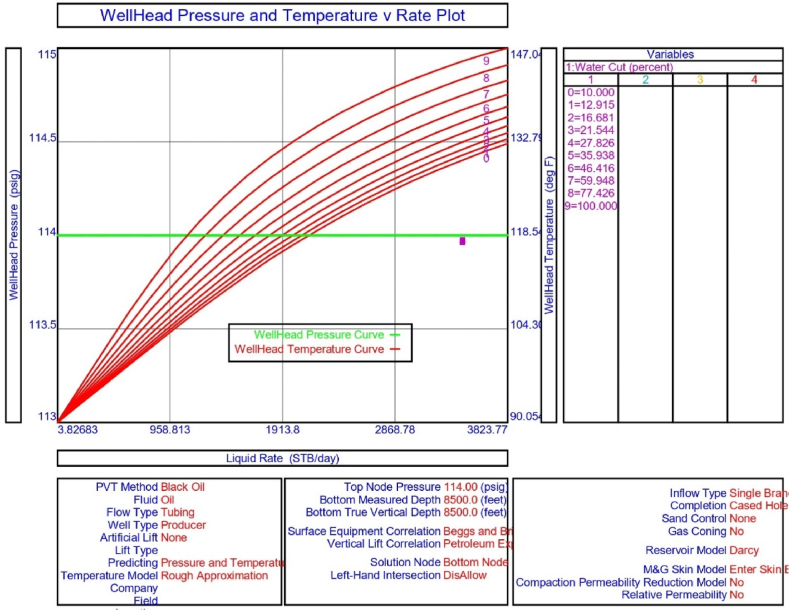
The effect of WHP and WHT on production flow rate for natural or self-flow well at different water cuts.

**Fig. 11:**
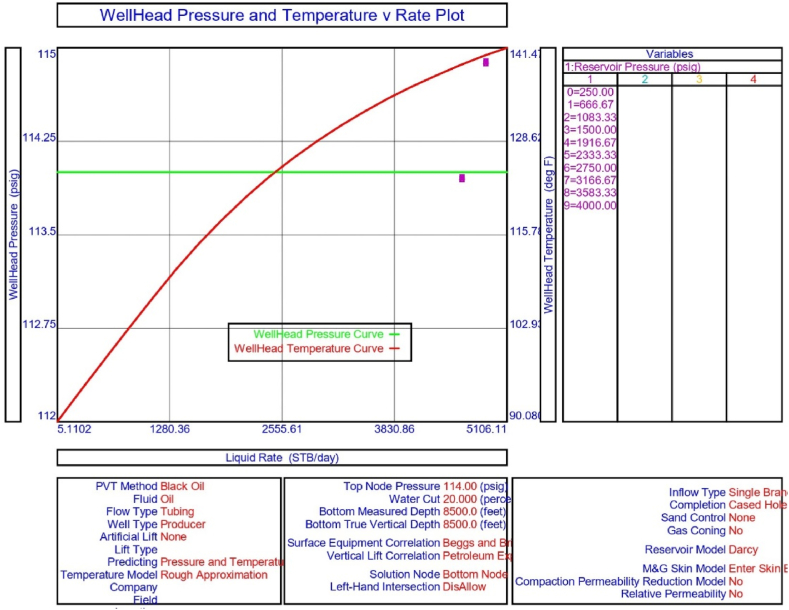
The effect of WHP and WHT on production flow rate for natural or self-flow well at different reservoir pressures.

**Fig. 12:**
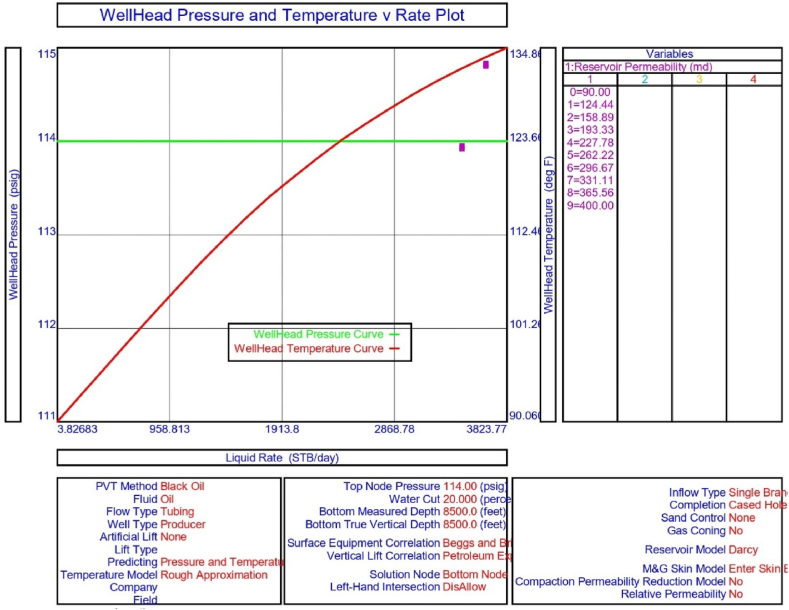
The effects of WHP and WHT on production flow rate for natural or self-flow well at different reservoir permeabilities.

**Fig. 13:**
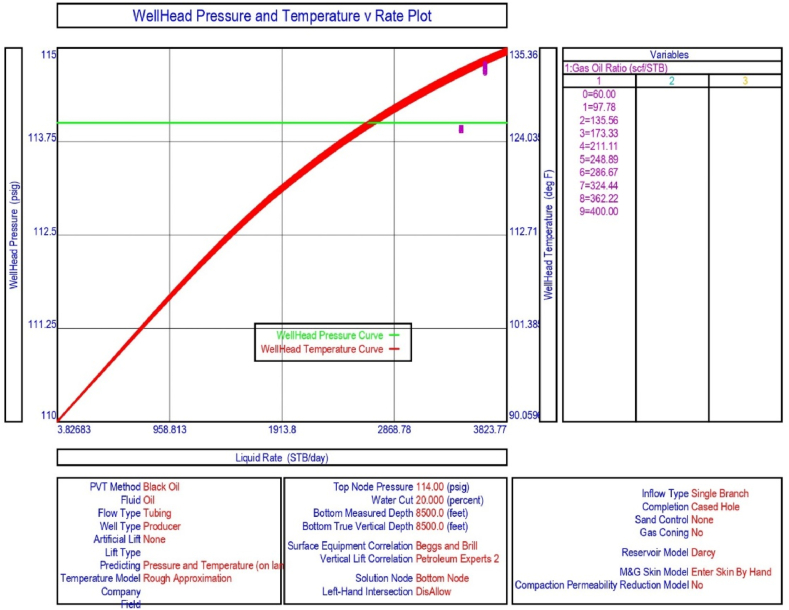
The effects of WHP and WHT on production flow rate for natural or self-flow well at different GORs.

**Table 5:** Sensitivity analysis on production flow rate of self-flowing well as a function of water cut.

Wellhead Pressure: 114 psig				
Water cut, %	Liquid rate, STB/day	Oil rate, STB/day	Water rate, STB/day	Wellhead Temperature, °F
10	362.8	326.5	36.3	95.20
12.9155	247.50	215.6	32.0	93.65
16.681	No flow	No flow	No flow	–
27.8256	No flow	No flow	No flow	–
35.9381	No flow	No flow	No flow	–
46.4159	No flow	No flow	No flow	–
59.9484	No flow	No flow	No flow	–
77.4264	No flow	No flow	No flow	–
100	No flow	No flow	No flow	–

**Table 6:** Sensitivity analysis on production flow rate of self-flow well as a function of reservoir pressure.

Wellhead pressure: 114 psig				
Reservoir pressure, psig	Liquid rate, STB/day	Oil rate, STB/day	Water rate, STB/day	Wellhead temperature. °F
250	0	0	0	0
666.667	No flow	No flow	No flow	–
1083.33	No flow	No flow	No flow	–
1500	No flow	No flow	No flow	–
1916.67	No flow	No flow	No flow	–
2333.33	No flow	No flow	No flow	–
2750	No flow	No flow	No flow	–
3166.67	489.7	391.7	97.9	97.74
3583.33	1068.5	854.8	213.7	106.75
4000	1573.6	1258.9	314.7	113.92

**Table 7:** Sensitivity analysis on production flow rate of self-flowing well as a function of reservoir permeability.

Wellhead pressure: 114 psig				
Reservoir permeability, md	Liquid rate, STB/day	Oil rate, STB/day	Water rate, STB/day	Wellhead temperature, °F
90	No flow	No flow	No flow	–
124.444	No flow	No flow	No flow	–
158.889	No flow	No flow	No flow	–
193.333	No flow	No flow	No flow	–
227.778	No flow	No flow	No flow	–
262.222	No flow	No flow	No flow	–
296.667	No flow	No flow	No flow	–
331.111	No flow	No flow	No flow	–
365.556	No flow	No flow	No flow	–
400	No flow	No flow	No flow	–

**Table 8:** Sensitivity analysis on production flow rate of self-flowing well as a function of GOR.

Wellhead pressure: 114 psig				
Gas Oil Ratio	Liquid rate, STB/day	Oil rate, STB/day	Water rate, STB/day	Wellhead temperature, °F
60	No flow	No flow	No flow	–
97.7778	No flow	No flow	No flow	–
135.556	No flow	No flow	No flow	–
173.333	No flow	No flow	No flow	–
211.111	No flow	No flow	No flow	–
248.889	553.0	442.4	110.6	98.78
286.667	795.6	636.5	159.1	102.66
324.444	1018.9	815.1	203.8	106.20
362.222	1224.5	979.6	244.9	109.37
400	1414.9	1131.9	283.0	112.20

**Fig. 14:**
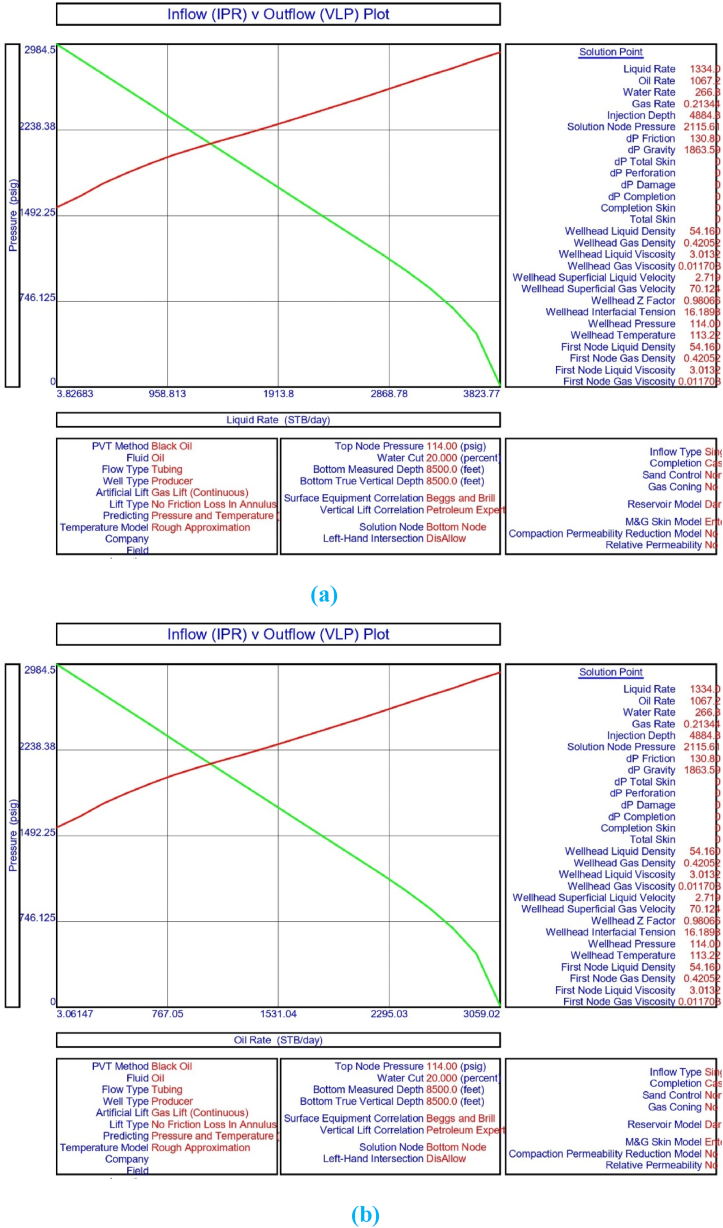
(a). IPR + VLP curve for a continuous flow gas lift (liquid rate). Fig. 14(b). IPR + VLP for a continuous flow gas lift (oil rate).

**Fig. 15:**
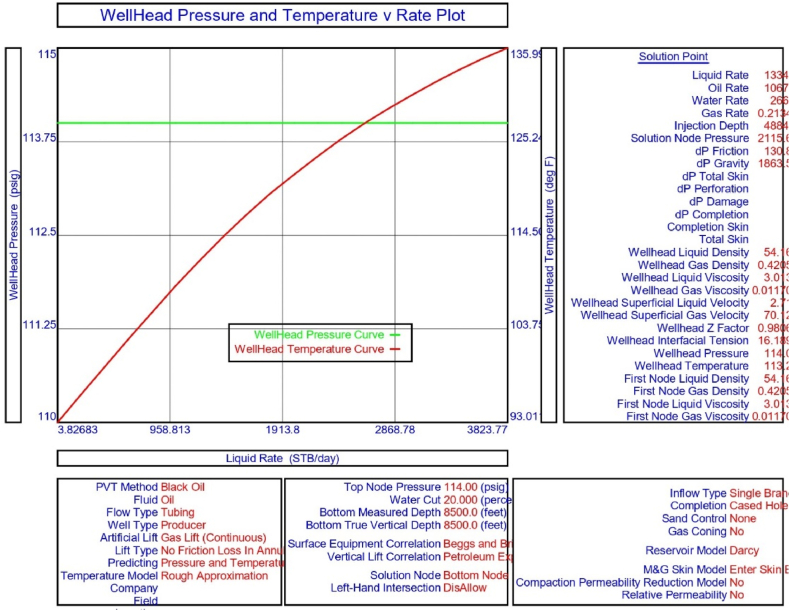
WHP and WHT versus production flow rate (liquid) for a continuous flow gas lift.

**Fig. 16:**
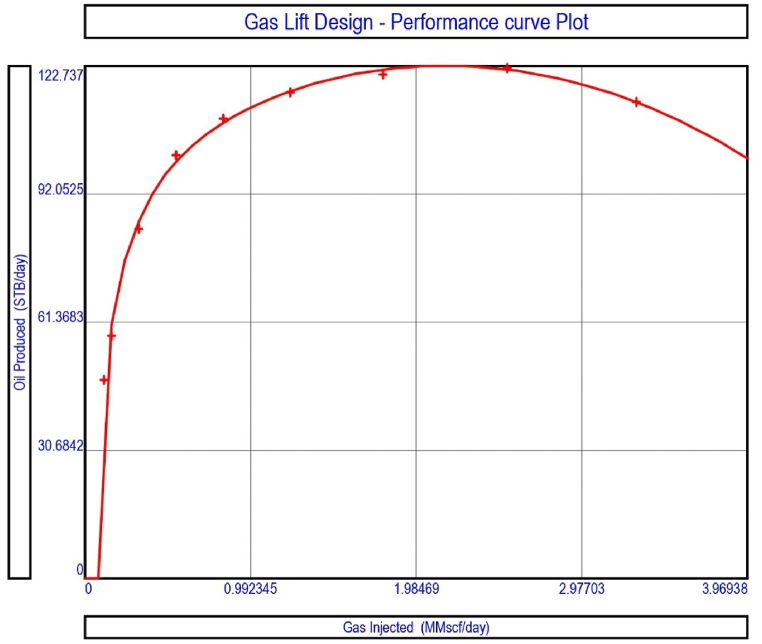
Gas lift design performance curve for a continuous flow gas lift.

**Fig. 17:**
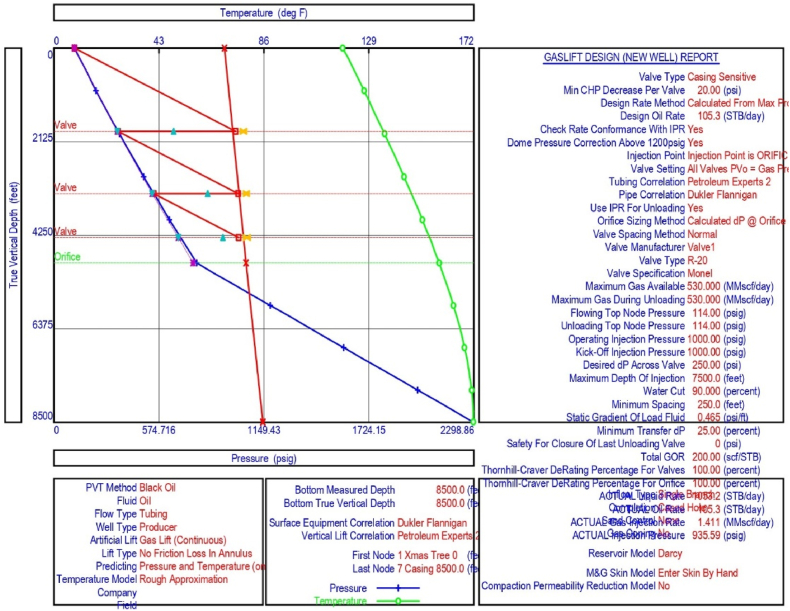
Gas lift design with valve setting positions for continuous flow gas lift.

**Fig. 18:**
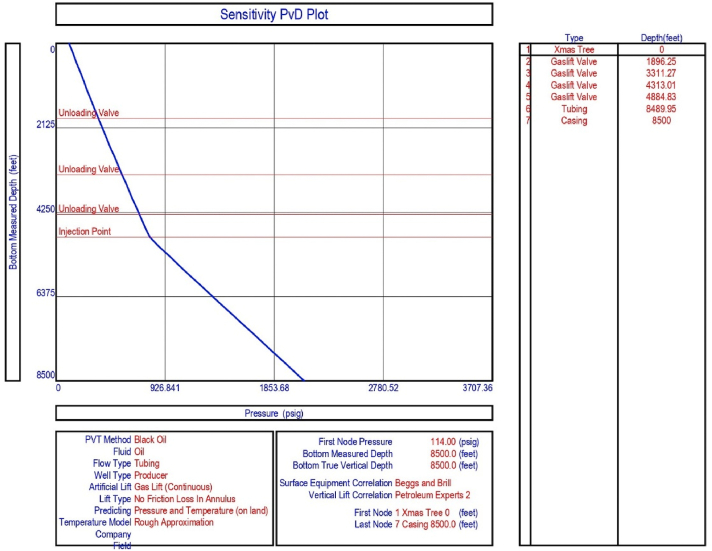
Valve setting depth for optimum gas injection in continuous flow gas lift.

**Fig. 19(a):**
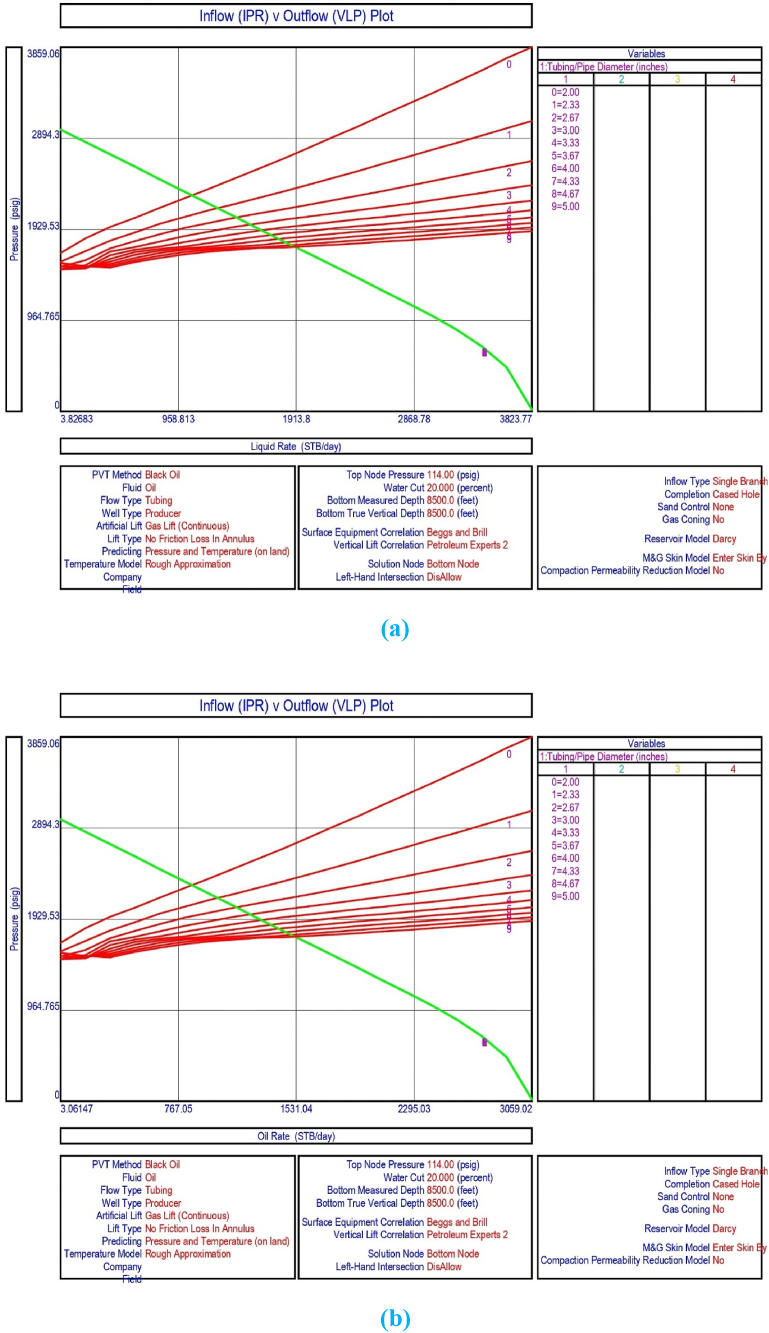
Sensitivity analysis on production flow rate considering inflow versus outflow performance curves for continuous flow gas lift as a function of tubing diameter (liquid rate). Fig. 19(b). Sensitivity analysis on production flow rate considering inflow versus outflow performance curve for continuous flow gas lift as a function of tubing diameter (oil rate).

**Fig. 20(a):**
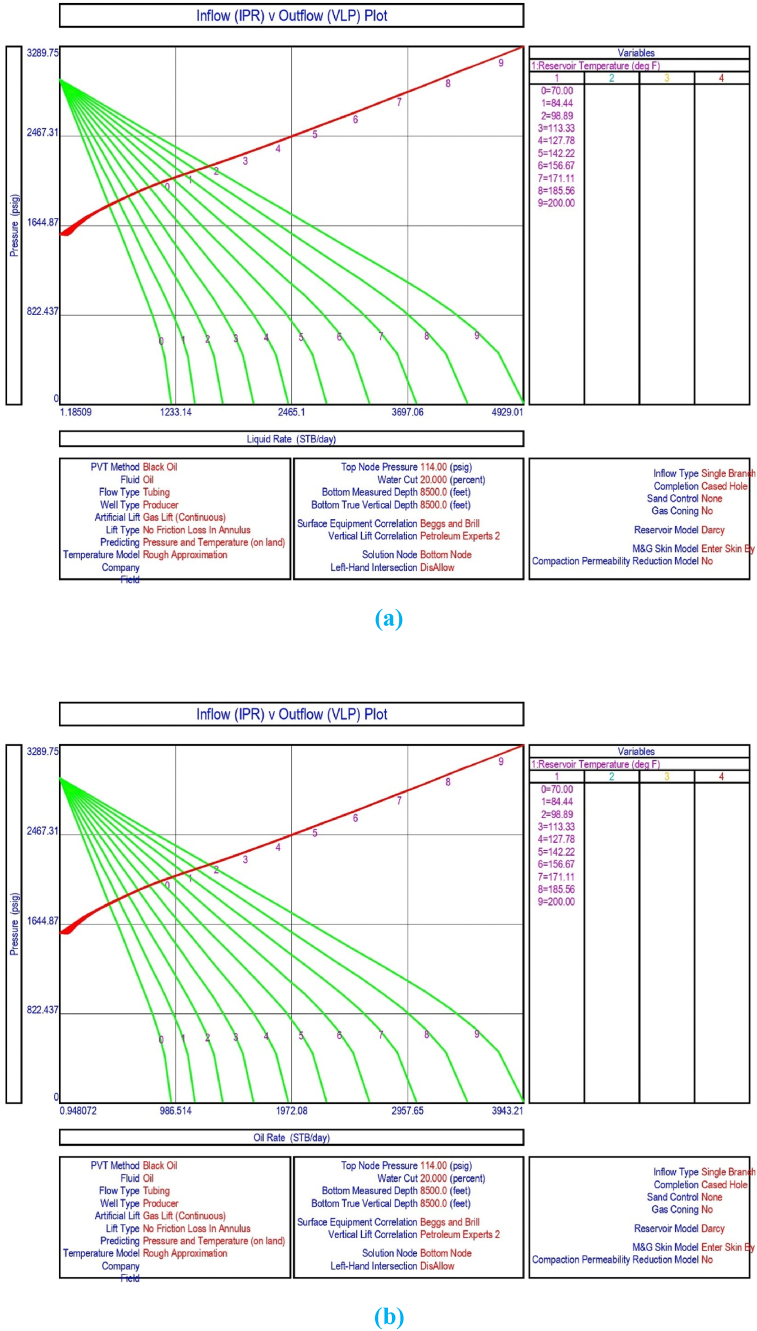
Sensitivity analysis on production flow rate considering inflow versus outflow performance curve for continuous flow gas lift as a function of reservoir temperature (liquid rate). Fig. 20(b). Sensitivity analysis on production flow rate considering inflow versus outflow performance curve for continuous flow gas lift as a function of reservoir temperature (oil rate).

**Fig. 21(a):**
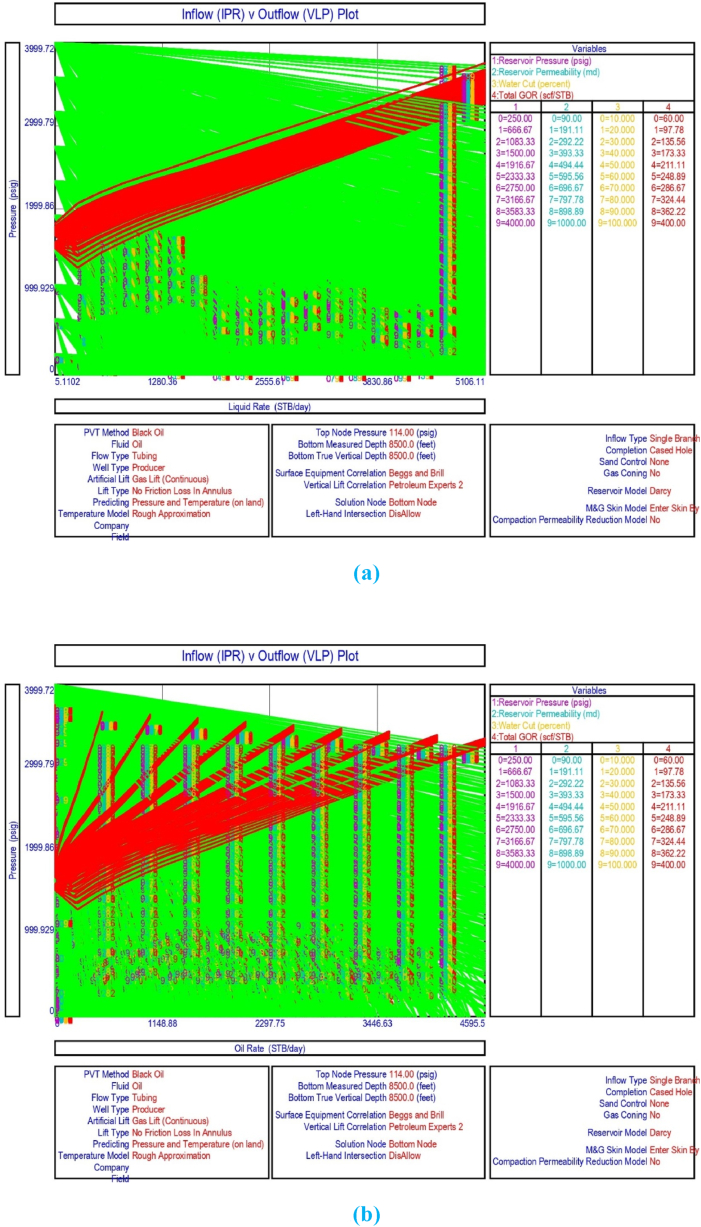
Sensitivity analysis on production flow rate considering the inflow versus outflow performance curve for continuous flow gas lift, as a function of reservoir pressure, permeability, water cut, and total GOR (liquid rate). Fig. 21(b). Sensitivity analysis on production flow rate considering the inflow versus outflow performance curve for continuous flow gas lift, as a function of reservoir pressure, permeability, water cut, and total GOR (oil rate).

**Fig. 22:**
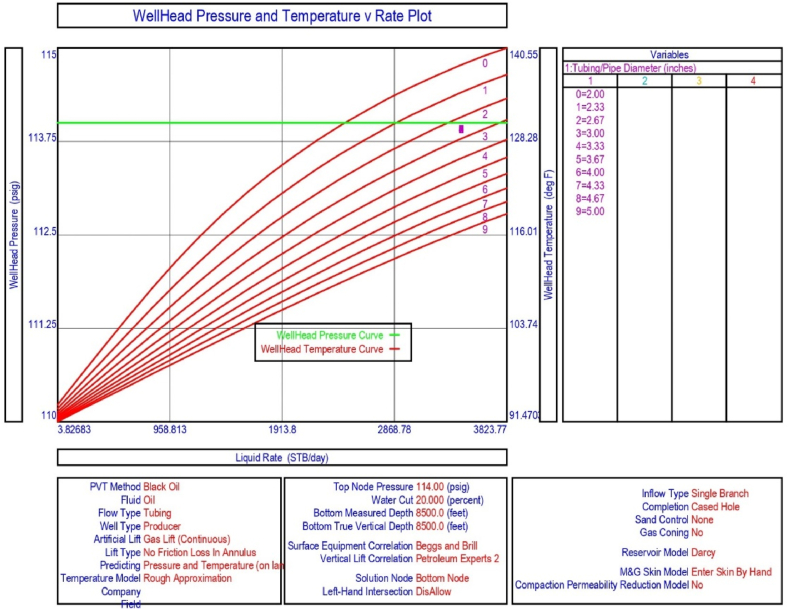
The effect of WHP and WHT on production flow rate in continuous flow gas lift as a function of tubing diameter.

**Fig. 23:**
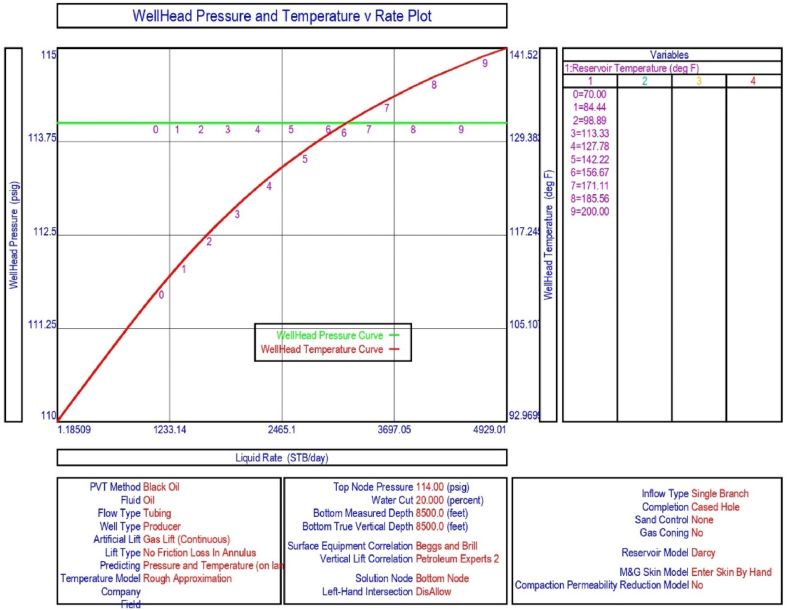
The effect of WHP and WHT on production flow rate in continuous flow gas lift as a function of reservoir temperature (liquid rate).

**Table 9:** Optimization of gas injection rate for continuous flow gas lift.

Injection rate (MMscf/day)	Liquid rate (STB/day)	Oil rate (STB/day)	Injection Pressure, psig
1.411	1053.2	105.3	935.59

**Table 10:** Sensitivity analysis on production flow rate as a function of tubing diameter for continuous flow gas lift.

Wellhead pressure: 114 psig				
Tubing diameter, inch.	Liquid rate, STB/day	Oil rate, STB/day	Water rate, STB/day	Wellhead temperature, °F
2	1073.0	858.4	214.6	113.43
2.3333	1280.4	1024.3	256.1	113.0
2.6667	1430.5	1144.4	286.1	112.68
3	1546.9	1237.6	309.4	111.71
3.3333	1639.2	1311.4	327.8	110.69
3.6666	1715.7	1372.6	343.1	109.67
4	1779.9	1423.9	356.0	108.71
4.3333	1833.4	1466.8	366.7	107.79
4.6667	1879.7	1503.8	375.9	106.93
5	1907.2	1525.8	381.4	106.05

**Table 11:** Sensitivity analysis on production flow rate as a function of reservoir temperature for continuous flow gas lift.

Wellhead pressure: 114 psig				
Reservoir temperature, °F	Liquid rate, STB/day	Oil rate, STB/day	Water rate, STB/day	Wellhead temperature, °F
70	531.1	424.9	106.2	101.33
84.4444	627.9	502.3	125.6	102.85
98.8889	729.5	583.6	145.9	104.43
113.3333	837.9	670.3	167.6	106.08
127.778	952.8	762.2	190.6	107.81
142.222	1073.0	858.4	214.6	109.57
156.667	1197.6	958.1	239.5	111.35
171.111	1326.0	1060.8	265.2	113.11
185.556	1456.2	1164.9	291.2	114.83
200	1586.6	1269.3	317.3	116.50

**Table 12:** Sensitivity analysis on production flow rate as a function of combined effect of reservoir pressure, permeability, water cut, and total GOR for continuous flow gas lift.

Reservoir pressure: 250 psig, reservoir permeability: 90 md, water cut: 10%, total GOR: 60 scf/bbl				
Reservoir pressure, psig	Liquid rate, STB/day	Oil rate, STB/day	Water rate, STB/day	Wellhead temperature, °F
1916.67	100.6	90.5	10.1	94.36
2333.33	216.3	194.7	21.6	95.99
2750	333.8	300.4	33.4	97.64
3166.67	452.8	407.5	45.3	99.31
3583.33	567.3	511.1	56.8	100.92
4000	680.4	612.3	68.0	102.49

The current work is based on production well data (i.e., well completion data), VLP, and IPR analysis. Reservoir rock and fluid analysis were part of the lab work. In this work, a detailed analysis of the reservoir model and its fine zonation and anisotropy behavior was not done as it was outside the scope of the work. Also, as these studies are not primary requirements in the PROSPER simulator, detailed information on these aspects is not available. Hence, these can be considered as limitations in the present work.

## 3 Results and discussions

### 3.1 Natural flow

Fig. 3 shows the result of the IPR plot for naturally flowing porous medium in the upper Assam basin. The findings of the system analysis utilising IPR and vertical lift performance (VLP) show that natural flow in the porous media is not occurring under current well circumstances, and the well has turned out to be a dead well because the intersection points between the IPR and VLP curves are missing (Fig. 4a and b). The current study hence performed sensitivity analysis to understand the effect of wellhead pressure, water cut, reservoir pressure, reservoir permeability, producing GOR, and tubing or pipe diameter on the flow condition of the well.

The integrated solution in the current study efficiently demonstrates the flowability of the oil well based on a set of actual conditions under which the oil well has to flow. When the multiple factors, i.e., wellhead pressure and temperature, water cut%, required reservoir pressure in the well to flow, gas injection rate, adequate available tubing diameter, GOR, and improvement to formation permeability, are taken into consideration, reliable findings on the required measures for revival of a dead well to production stage can be reached.

In this work, data from deviation surveys and the well's downhole completion were used to model the porous media under natural flow, as shown in Fig. 5. The result of the sensitivity analysis for the water cut is represented by Fig. 6 (a) (b), the reservoir pressure by Fig. 7 (a) (b), the reservoir permeability by Fig. 8 (a) (b), and the GOR by Fig. 9 (a) (b). In the subsequent analysis, the effects of wellhead pressure (WHP) and wellhead temperature (WHT) at various water cuts (Fig. 10), reservoir pressure (Fig. 11), reservoir permeability (Fig. 12), and GOR (Fig. 13) were investigated. Table 5, Table 6, Table 7, Table 8 demonstrate the sensitivity analysis for water cut, reservoir pressure, reservoir permeability, and GOR.

The current study replicates the natural flow of a porous medium in PROSPER simulation software by taking into account PVT data for reservoir fluids. In the present analysis, the IPR data was correlated with downhole information such as measured depth (MD) and true vertical depth (TVD). Following this, input data from the formation at a measured depth of 0–8500 ft was taken into account. In the current analysis, the geothermal gradient values of 90 °F and 172 °F were included for the extreme end of the well depth. The overall heat transfer coefficient was set to 8 BTU/h/ft^2^/°F. The Black Oil Model was used in this study, with oil and water as the flowing fluids, and no gas lift system was included in the model study. Table 1, Table 2, Table 3, Table 4 illustrate the data for modelling the naturally flowing well. After appropriate matching of PVT data, Darcy's model was used to build the IPR in the current investigation (Fig. 3). Mechanical/geothermal skin was also chosen for manually adding the necessary information. As demonstrated in Fig. 4 (a) and 4 (b), the well is a dead well that cannot flow naturally. This was established by the lack of an intersection point between the IPR and VLP curves for porous media under natural flow. The IPR curve in Fig. 3 predicted the absolute open flow potential (AOF) to be 3826.8 STB/day and the PI to be 1.53 STB/day/psi for the current investigation. The next step was to conduct an assessment to determine what was causing the lack of flow in the well.

The sensitivity analysis results for inflow and outflow performance curves at various water cuts, as illustrated in Fig. 6 (a) and 6 (b) and further detailed in Table 5, reveal that when the water cut increases by 16.681%, the well loses its capacity to flow naturally. In addition, the crude oil production requires a consistent supply of gas lift gas as the well under analysis is currently producing with a 20% water cut. The effect of WHP and WHT was explored in the current configuration, and the system analysis in PROSPER reveals that the liquid production rate as referred to in Fig. 10 declines with increasing water cut at a node pressure of 114 psig.

The porous media was then assessed using IPR and VLP curve analysis to calculate flow potential at different reservoir pressures and the results are shown in Table 6. Following this, the IPR and VLP curve analysis were used for performance evaluation of the porous media in obtaining flow potential at different reservoir pressures. The analysis of the porous medium prior to the installation of the gas lift, as illustrated in Fig. 7 (a) and (b), reveals that the well's flowability is not achievable with the current reservoir pressure of 250 psig. This may be determined via sensitivity analysis at various reservoir pressures, which demonstrates that the wells achieve start-up of fluid flow as the pressure rises from 250 psig to 3166.67 psig. However, in order to maintain production, the well may need to be put on gas lift or subjected to secondary recovery procedures such as water flooding. Similarly, the WHP and WHT analysis on the dead porous medium results in Fig. 7(a) and (b), and 11 imply that if a needed pressure of 3166.67 psig can be made available for the well in the current study, a node pressure of 114 psig and a WHT of 97.74 °F may result in crude oil production from the well (Table 6).

Following that, the effect of reservoir permeability on the dead well was explored. The sensitivity analysis findings for IPR and VLP curves at various permeability values, as shown in Fig. 8 (a) and 8 (b), infer that the porous medium cannot flow with the existing formation permeability of 300 md. There is no way that increasing permeability to 400 md will result in crude oil production. According to the sensitivity assessment in Table 7, there will be no oil production in the upper Assam basin's porous medium under current conditions.

However, the impact of different GORs on liquid and oil production rates was obvious in the results reported in Fig. 9 (a) and (b). According to the sensitivity analysis with IPR and VLP curves displayed in Fig. 9 (a) and (b) and related data supplied in Table 8, the existing GOR (200 scf/STB) for the well in the current study does not support the flow of crude oil in the porous media. To keep the well flowing, the GOR can be increased from 200 to 248.889 and beyond (Table 8). This finding reinforces the need for continuous flow gas lift installation in porous materials.

### 3.2 Gas lift

This section depicts the impact of adding a continuous flow gas lift system to a dead well that was not flowing under natural flow. The effectiveness of the continuous flow gas lift system is shown in Fig. 14 (a) and **(b)**, which take into account the inflow and outflow performance curves. Key PROSPER data, such as reservoir and PVT data, as well as productivity index values for porous media with gas lift installation, were factored into the simulation results. Based on the intersection of the IPR and VLP curves, the flowability that can be achieved in porous media under continuous gas lift at a certain pressure and liquid rate is demonstrated in Fig. 14 (a) and (b). The outcome shown in Fig. 14 (a) and (b) ensures that oil production will start after the installation of a continuous flow gas lift. The associated outflow performance curves for wellhead pressure (WHP) and temperature (WHT), as illustrated in Fig. 15, were then examined to see how they affected the liquid flow rate in the well under analysis. The results of the ensuing assessment of gas lift performance are examined and displayed in Fig. 16. In this case, Table 9 indicates the relevant optimum gas injection rate. The findings for unloading and operating valves are presented in Fig. 17, Fig. 18. The current study investigated the valve setting depths after optimising the gas lift. The results of the sensitivity analysis performed using the IPR and VLP performance analysis are shown in Fig. 19 (a) (b), 20 (a) (b), and **21 (a) (b)**, which exhibit the effects of tubing diameter, reservoir temperature, and combined effects of variables, namely water cut, reservoir permeability, total GORs, and reservoir pressure, respectively. Table 10, Table 11, Table 12 demonstrate the sensitivity analysis for tubing diameter, reservoir temperature, and the combined effects of reservoir permeability, water cut, and GOR. In the subsequent analysis, the effects of wellhead pressure (WHP) and wellhead temperature (WHT) at various tubing diameters (**Fig. 22**) and reservoir temperature (**Fig. 23**) are presented.

Continuous gas injection resulted in the restart of production from a dead well, as shown in Fig. 14(a) and (b). Following the initiation of gas injection, the IPR and VLP curves are now intersecting at a given pressure and liquid rate. Table 9 presents the gas lift design for the current investigation, which highlights the optimization of gas injection rate for efficient recovery from oil wells. Recovery is possible following continuous gas lift installation due to a decrease in oil density in the tubing with gas injection, resulting in a reduction in hydrostatic head by placing the unloading and operating valves at the proper depths and injecting the proper amount of gas as estimated by the PROSPER software. As illustrated in Fig. 15, the requisite combination of wellhead pressure and temperature is also sufficient for the fluid to flow.

After the simulation result with gas injection, the gas lift performance curve was processed in PROSPER. The result given in Fig. 16 shows that when the gas injection rate enhances, oil production improves steadily until it reaches a peak of around 1.98469 MMSCf/day, after which it starts to fall. In the current investigation, different injection rates ranging from 0 to 3.96938 MMScf/day were examined. In comparison to the measured or calculated gas injection rate of 2.163 MMScf/day, the current analysis find that the optimum gas injection rate of 1.41135 MMScf/day with an injection pressure of 935.589 psi can be employed to recover crude oil. Table 9 shows the results of the sensitivity analysis for the gas injection rate. The use of optimal gas injection not only maintains long flowability, but it also lowers a condition known as "gas slippage” between oil and gas, in which gas flows quicker than oil, causing oil production to decrease [7]. In this study, the optimal injection gas rate for oil production of 105.323 STB/day and a liquid rate of 1053.23 STB/day was determined to be 1.41135 MMScf/day (Table 9).

The current analysis also indicated the need for three unloading valves for start-up operation at depths of 1896.25 ft, 3311.27 ft, and 4313.01 ft, as well as an operational valve for gas injection at a depth of 4884.83 ft. Fig. 17, Fig. 18 demonstrate the results.

Following that, sensitivity analysis for various tubing sizes was carried out using the IPR and VLP analytic tools in order to investigate the effects on crude oil production from porous media in the upper Assam basin. The result displayed in Fig. 19 (a) shows that the liquid flow rate (STB/day) can be increased by increasing the tubing diameter. A higher production rate is achieved using larger diameter tubing (Table 10). As shown in Fig. 19 (a) and 20 (b) and Table 10, the IPR and VLP curves intersect for all tubing diameters from 2.0 inches to 5 inches, ensuring oil production as tubing diameter increases. Furthermore, given the present nodal pressure of 114 psi, tubing diameters of 2.0–3.0 inches will suffice for fluid production, with the largest size being 3 inches, contributing to maximum output (Fig. 19). However, as illustrated in Fig. 19, increasing tubing diameter further will not meet the required wellhead temperature, resulting in a reduction in crude oil production.

Sensitivity analysis of reservoir temperature effects on liquid production rate conducted using IPR and VLP curves shows that when reservoir temperature rises, the liquid production rate from the well increases as well. This was determined by PROSPER analysis and is shown in Fig. 20 (a) and (b), as well as Fig. 23 and Table 11.

The combined effects of reservoir pressure, reservoir permeability, water cut, and total GOR were studied in this work, and the results are given in Fig. 21 (a) and 21 (b) and Table 12. In this study, reservoir pressures were changed, and the associated oil or liquid production rate capability was examined with increasing water cut, as indicated in Table 12. According to this comparison analysis, using a continuous flow gas lift can result in a higher crude oil production rate of up to 680.4 STB/day with a water cut of up to 68%. For the well to flow, however, a minimum of 1916.67 psi reservoir pressure support is required (Table 12).

## 4 Conclusion

The current work on sandstone porous medium, which is connected to a dead well with a 20% water cut, was investigated in order to understand the conditions that could be hindering the oil well from flowing. The pressure and temperature changes along the tubing while fluid flows from the intake to the top of the tubing were considered in this work with PROSPER. However, external thermal fluid injection was not covered in this study. PROSPER is a reliable tool to design and model well completion based on simulation work related to fluid characterization, VLP analysis, and reservoir inflow performance studies. The simulator running time for sensitivity analysis is performed by the system, for which customised running timing is not required. This helps in getting reliable results with PROSPER. The present work deals with the oil well production issue that was producing naturally. The scope of the work assumes a single zone of uniform porosity and permeability for simplification of the model, as details on the reservoir model were unavailable. The anisotropies have not been considered within the scope of the work. The current work focuses mainly on well data to address and overcome the primary issue of low reservoir pressure that is obstructing fluid flow.

According to the model analysis performed using PROSPER simulation software, low reservoir pressure was the primary reason for the well becoming a dead well. Further examination of the water cut finds that water breakthrough results in a non-flowing state in the well at the current nodal pressure of 114 psi due to the current low pressure. In this case, a reservoir pressure of at least 3166.67 psig is required for the well to flow without the need for a gas lift (Table 6). Because the well's reservoir pressure is low, it must be put on artificial lift as soon as feasible, or else pressure maintenance operations or water floods will be required. Given the lack of formation damage in the well, it is evident that no amount of formation damage improvement will be able to establish flow in the downhole. When GOR was increased from its existing value of 200–248.889 scf/STB, the system responded favourably to flow (Table 8). As a result, this finding lends credence to the use of gas injection to resurrect the dead well. According to the current investigation, continuous flow gas lift could be installed under such low pressure well conditions. This was demonstrated by the crossing points that formed between the IPR and VLP graphs when gas was injected into the well. Gas injection can increase oil output by lowering the oil density in tubing and lowering the overall hydrostatic pressure by allowing the well to flow at the existing reservoir pressure. However, an excessive increase in gas injection rate beyond the optimal gas injection rate of 1.41135 MMScf/day in the current study on the oil-producing porous medium in the upper Assam basin would result in gas slippage and hence a decrease in oil production rate. Oil recovery is also influenced by the depth of injection gas or the operating valve, with 4884.83 feet being the optimal depth for the well. Furthermore, the larger the tubing, the higher the liquid flow rate, given that the wellhead pressure is low enough to allow fluid to flow up the tubing. In the production of crude oil, the temperature of the reservoir is also significant. The rate of production increases as the temperature of the reservoir rises. Finally, with a reservoir pressure of 1916.67 psi maintained, the well will be able to produce with a water cut value of up to 10% under continuous flow gas lift installation (Table 12). Finally, the current study finds that a dead well with low to medium reservoir pressure and no formation damage can be coaxed to flow again by using continuous flow gas lift. Furthermore, this novel study examines tubing diameter, reservoir pressure, reservoir temperature, water cut, and GOR, all of which were found to have an impact on crude oil production. When faced with early water breakthrough, wells with low reservoir pressure have limited flow potential, and the well may become a dead well. The larger the tubing diameter, the greater the increase in crude oil production, provided that wellhead pressure and temperature do not drop excessively, resulting in gas breakthrough and increased oil viscosity. The higher the reservoir temperature, the greater the production of crude oil from a continuous flow gas lift well. To produce a well with increasing water cut under continuous flow gas lift well, it is required to have a higher reservoir pressure. During the optimization process, the rate of gas injection, as well as the position of gas injection and the injection pressure, are all crucial well parameters to consider. Furthermore, wellhead pressure has an impact on the optimization technique for gas lift.

## Author contribution statement

Dhrubayoti Neog: Conceived and designed the experiments; Performed the experiments; Analyzed and interpreted the data; Contributed reagents, materials, analysis tools or data; Wrote the paper.

## Data availability statement

Data included in article/supp. material/referenced in article.

## Additional information

No additional information is available for this paper.

## Declaration of competing interest

The author declares that he has no competing interests of any kind.

## Nomenclature

API: American Petroleum Institute
AOF: Absolute open flow
B/D: Barrel Per Day
BTU/hour/ft^2^: British thermal unit per *hour* per square foot
ESP: Electrical Submersible Pump
GL: Gas Lift
GOR: Gas/oil ratio
GLR: Gas-liquid ratio
H: Formation thickness
IPR: Inflow Performance Relationship
K_x_, K_y_, K_z_: Effective permeability
MD: Measured Depth
MMscf/day: Million standard cubic feet
PCP: Progressive Cavity Pump
PROSPER: Productions and Systems Performance Analysis System
PVT: Pressure-Volume-Temperature
PI: Productivity Index
Psig: Pounds per square gauge
RT: Rock Type
SRP: Sucker Rod Pump
SCF/bbl: Standard cubic feet of Natural Gas per barrel of crude oil at stock tank conditions
STB/day: Stock tank barrel/day
TPR: Tubing Performance Relationship
TVD: True Vertical Depth
VLP: Vertical Lift Performance Relationship
WHP: Wellhead Pressure
WHT: Wellhead Temperature
ppm: *parts* per *million*
pH: potential of hydrogen
md: millidarcy
φ: Porosity
ρ: Density
P_b_: Bubble Point Pressure
R_s_: Gas solubility
β_o_: Oil formation volume factor
in: inch
ft: foot or feet
